# The Roles of MiRNAs (MicroRNAs) in Melanoma Immunotherapy

**DOI:** 10.3390/ijms232314775

**Published:** 2022-11-25

**Authors:** Linyinxue Dong, Xuechen Tian, Yunqi Zhao, Haohong Tu, Aloysius Wong, Yixin Yang

**Affiliations:** 1 Wenzhou Municipal Key Laboratory for Applied Biomedical and Biopharmaceutical Informatics, Wenzhou-Kean University, Wenzhou 325060, China; 2 Zhejiang Bioinformatics International Science and Technology Cooperation Center, Wenzhou-Kean University, Wenzhou 325060, China; 3 Department of Biology, College of Science and Technology, Wenzhou-Kean University, 88 Daxue Road, Ouhai, Wenzhou 325060, China; 4 School of Natural Sciences, Dorothy and George Hennings College of Science, Mathematics and Technology, Kean University, 1000 Morris Ave, Union, NJ 07083, USA

**Keywords:** miRNA (microRNA), melanoma, immunotherapy, immune checkpoint inhibitor

## Abstract

Melanoma is the most aggressive form of skin cancer, characterized by life-threatening and rapidly spreading progression. Traditional targeted therapy can alleviate tumors by inactivating hyperactive kinases such as BRAF or MEK but inevitably encounters drug resistance. The advent of immunotherapy has revolutionized melanoma treatment and significantly improved the prognosis of melanoma patients. MicroRNAs (miRNAs) are intricately involved in innate and adaptive immunity and are implicated in melanoma immunotherapy. This systematic review describes the roles of miRNAs in regulating the functions of immune cells in skin and melanoma, as well as the involvement of miRNAs in pharmacology including the effect, resistance and immune-related adverse events of checkpoint inhibitors such as PD-1 and CTLA-4 inhibitors, which are used for treating cutaneous, uveal and mucosal melanoma. The expressions and functions of miRNAs in immunotherapy employing tumor-infiltrating lymphocytes and Toll-like receptor 9 agonists are also discussed. The prospect of innovative therapeutic strategies such as the combined administration of miRNAs and immune checkpoint inhibitors and the nanotechnology-based delivery of miRNAs are also provided. A comprehensive understanding of the interplay between miRNAs and immunotherapy is crucial for the discovery of reliable biomarkers and for the development of novel miRNA-based therapeutics against melanoma.

## 1. Introduction

Melanoma is one of the most common cutaneous cancers resulting from the oncogenic aberration of melanocytes in the skin of various parts of the human body such as legs, back, head and neck, as well as in the eyes and mucosa tissues of other organs [1]. The incidence and mortality of melanoma have been increasing every year. In 2020, 324,635 new cases of cutaneous melanoma were diagnosed, and 57,043 new deaths were reported worldwide. In other words, about one person is diagnosed with melanoma every two minutes, and more than one person dies from this disease every ten minutes [2]. It is worth noting that melanoma tumorigenesis exhibits significant discrepancies in different ethnic populations. For instance, compared with black and Asian populations, white people are far more likely to be inflicted with melanoma [3]. Melanoma can be successfully treated if diagnosed at an early stage; however, if metastasis occurs, it responds poorly to conventional anti-cancer therapies, as reflected by the poor 5-year survival rate of only 15–20% [4].

The advent of molecular targeted therapies and immunotherapy has significantly improved the treatment of malignant melanoma. Molecularly-targeted therapies inhibit the aberrant activation of the mitogen-activated protein kinases (MAPK) signaling pathway caused by hyperactive mutations in the proto-oncogene BRAF, encoding for a serine/threonine kinase that upregulates the RAS/RAR/MEK pathway. Several BRAF kinase inhibitors, such as Vemurafenib, Dabrafenib, and Encorafenib, which target BRAFV600E or BRAFV600K, have been used to treat advanced malignant melanoma [5]. By releasing immune checkpoint blockage such as cytotoxic T-lymphocyte-associated antigen 4 (CTLA4), and the programmed cell death protein 1 (PD-1) and its ligand (PD-L1), anti-tumor immune responses can be reactivated. Immunotherapy represents the most promising therapeutic strategy against malignant melanoma, as it can achieve a much more sustained therapeutic effect than targeted therapies [6,7]. Targeted therapies and immunotherapy have achieved some success, but the inevitable resistance to targeted therapies and the side effects of immunotherapy remain an intricate issue [8,9,10].

**Figure 1:**
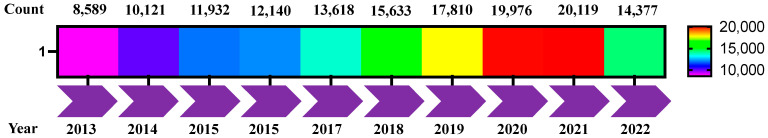
Number of miRNA related publications in the past ten years (data obtained from PubMed).

MicroRNAs (miRNAs) were first discovered in 1993 as a cluster of non-coding RNAs with a length of 21–23 nucleotides [11,12]. Since then, miRNA has become a prominent research area. The number of miRNA-associated scholarly articles has been rapidly increasing in the past ten years (Figure 1), which reflects the critical roles of miRNA in various biological processes and diseases including cancers. In the latter, numerous research has studied the gene expression levels of miRNAs in melanoma and based on their tumor promoting or inhibiting properties, they are classified as oncogenes or onco-suppressors [13]. Moreover, their roles in the initiation, development and metastasis of melanoma have also been studied [14]. At the molecular level, scientists have resolved the regulatory mechanisms of miRNAs including their target genes and signaling pathways [15]. Importantly, the expression levels of miRNAs have been clinically linked to the response rate, efficacy and side effects of the treatments [16,17]. Meanwhile, due to the differential expression of miRNAs in the serum and other body fluids of melanoma patients, miRNAs have been explored as potential prognostic biomarkers [18]. However, the in-depth mechanisms of how miRNAs are associated with the immune system have not been comprehensively reviewed. The combined administration of miRNAs-based drugs and other anticancer agents are currently under investigation, which could potentially provide an effective therapeutic strategy for melanoma treatment. In this article, we provide a systematic review of the latest research and potential applications of miRNAs in melanoma, especially the roles of miRNAs in tumor immunity and melanoma immunotherapy. We also present the latest developments of miRNA-based lipid drugs and drug delivery carriers.

## 2. Cutaneous Melanoma

Among all forms of cancers, cutaneous melanoma is a malignancy that is best studied and understood within the context of miRNAs. This section provides an insight into the regulation and interaction of miRNAs with the innate and adaptive immune systems, as well as the roles of miRNAs in immunotherapies.

### 2.1. MiRNAs in Cutaneous Melanoma

**Figure 2:**
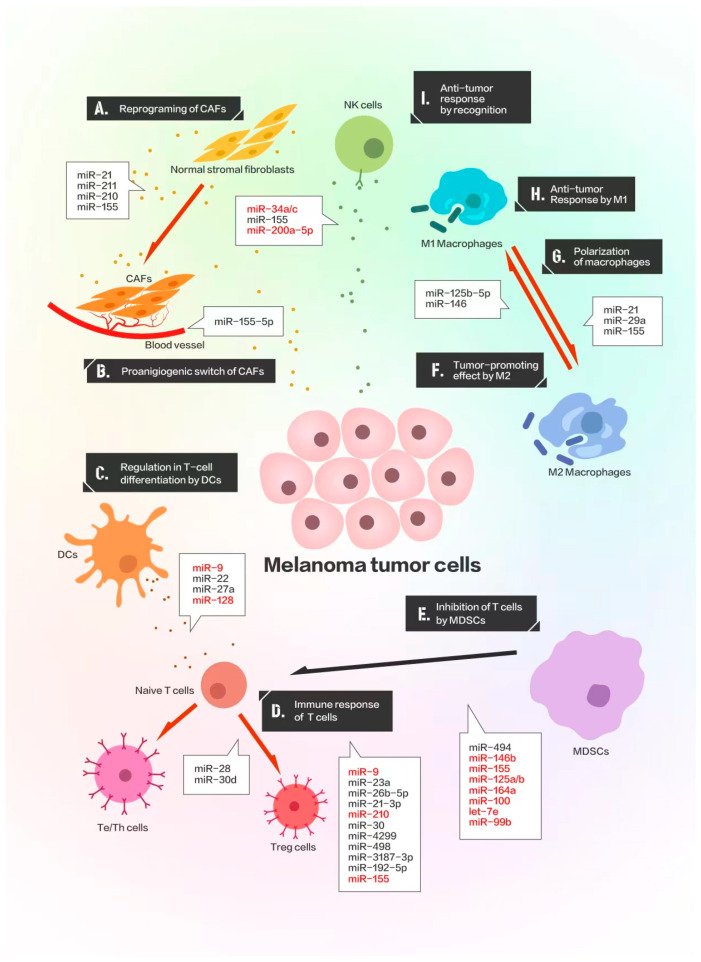
The roles of miRNA in the innate and adaptive immune systems. The miRNAs in black are oncogenic while those in red are tumor-suppressive.

The innate immunity underlies the initial responses against tumor cells with the participation of cancer-associated fibroblasts (CAFs), dendritic cells (DCs), macrophages, and natural killer cells (NKs). The roles of specific miRNAs in different immune cells are illustrated in Figure 2 while the functions and target genes of miRNAs engaging in immune responses of the different immune cells are summarized in Table 1.

**Table 1:** Functions and interaction of MiRNAs in immune cells of melanoma.

Upstream Stimulation	MiRNAs	Downstream Regulation	Functions	Experimental Methods	Literatures
**Cancer-associated fibroblasts (CAFs)**					
TGF-β1 treatment	miR-21 ↑	Direct target: SMAD7 ↑ TGF-β signaling ↑ FSP1 expression ↑	Formation of CAFs	In melanoma cell lines + murine models	[19]
/	miR-211 ↑	Direct target: IGF2R expression ↓ MAPK signaling ↑ Pro-inflammatory genes: IL1-β, IL-6, IL-8, CXCL1 and CXCL2 expression ↑	Formation of CAFs	In melanoma cell lines + murine models	[20]
/	miR-210 and miR-155 ↑	Glycolysis metabolism ↑ Oxidative phosphorylation ↓	Formation of CAFs	Only in melanoma cell lines	[21]
/	miR-155-5p ↑	Direct target: SOCS1 ↓ JAK2/STAT3 signaling ↑ Proangiogenic factors: VEGFa, FGF2, and MMP9 ↑	Formation and the proangiogenic switch of CAFs	In melanoma cell lines + murine models	[22]
**Dendritic cells (DCs)**					
Pro-inflammatory stimulation of lipopolysaccharide (LPS)	miR-9 ↑	Direct target: PCGF6 ↓ Pro-inflammatory cytokines: IL-12p40, IL-6, TNFa, and IL-12p70 ↑	Increasing activation of T cells	In melanoma cell lines + murine models	[23]
/	miR-22 ↑	Direct target: p38 ↓ MAPK signaling ↓ IL-10 expression ↓	Inhibiting Th17 cells differentiation	In melanoma cell lines + murine models	[24]
TGF-β treatment	miR-27a ↑	Direct target: TAB3, p38 MAPK, MAP2K4 and MAP2K7 ↓ NF-kB and MAPK–JNK signaling ↓ (NF-a, IL-1b, IL-6, IL-12 and IL-23 ↓)	Inhibiting the differentiation of Th1 and Th17 cells	In melanoma cell lines + murine models	[25]
/	miR-128 ↑	Direct target: p38 ↓ MAPK signaling ↓ (Expression of IL-6 and IL-10 ↑, IL-12 ↓)	Enhancing anti-tumor ability of DCs	In melanoma cell lines + murine models	[26]
**Macrophages**					
/	miR-21 ↑	Direct target: STAT1 ↓ IFN-γ-induced STAT1 signaling ↓	Inhibiting polarization of M1 macrophages and decreasing the anti-tumor ability of macrophages	In melanoma cell lines + murine models	[27]
/		GZMB, CXCL10, IL-10 ↓	Decreasing cytotoxicity of CD8^+^ T cells and improving angiogenesis of melanoma cells	In melanoma cell lines + murine models	[28]
Activation of CSF1-ETS2 pathway	miR-21 and miR-29a ↑	miR-21 targets: Pdcd4, Spry1 and Timp3 ↓ miR-29a targets: Col4a2, Sparc and Timp3 ↓	Inhibiting polarization of M1 macrophages and promoting tumor cell proliferation and angiogenesis	In melanoma cell lines + murine models	[29]
/	miR-125b-5p ↑	Direct targets: LIPA ↓ (CCL1, CCL2, and IL-1β expression ↑)	Reinforcing the polarization of M1 macrophages and prolonging the survival of macrophages	Only in melanoma cell lines	[30,31,32]
Activation of NF-κB signaling	miR-146 ↑	TRAF6, IRAK1, and IRAK2An NF-kappaB ↓	Promoting polarization of M1 macrophages and immune response of macrophages	In melanoma cell lines + murine models	[30,33,34]
/	miR-155 ↑	IFN-inducible genes ↑	Promoting the activation of M2 macrophages	In melanoma cell lines + murine models	[35,36,37]
**Natural killer cells (NKs)**					
/	miR-34a/c ↑	ULBP2 expression ↓ NKG2D-dependent signaling ↓	Promoting the recognition and suppression of tumors in NK cells	Only in melanoma cell lines	[38]
/	miR-155 ↑	IFN-c and granzyme B production and NKG2D expression ↑	Decreasing the sensitivity of melanoma cells to NK cells cytolysis	Only in melanoma cell lines	[39]
/	miR-200a-5p ↑	Direct target: TAP1 expression ↓ HLA-I pathway ↓	Increasing the NK cells recognition to melanoma cells	Only in melanoma cell lines	[40]
**Myeloid derived suppressor cells (MDSCs)**					
Induction of TGF-β 1	miR-494 ↑	Direct target: PTEN ↓ Akt pathways ↑ Levels of metalloproteinases (MMP2, MMP13, and MMP14) ↑	Increasing the activity of MDSCs as well as tumor cell invasion and metastasis	In melanoma cell lines + murine models	[41]
/	miR-146b, miR-155, miR-125a/b, miR-164a, miR-100, let-7e and miR-99b ↑	STAT3 downstream signaling pathways ↓	Polarization and T cells-inhibiting functions of MDSCs	In melanoma cell lines + murine models	[18]
**T cells**					
Pro-inflammatory stimulation of lipopolysaccharide (LPS)	miR-9 ↑	Direct target: PCGF6 ↓ pro-inflammatory cytokines IL-12p40, IL-6, TNFa, and IL-12p70 ↑	Increasing antigen sensitivity of CD8^+^ OT1 T cells	In melanoma cell lines + murine models	[23]
TCR activation and TGF-β signaling	miR-23a ↑	Direct target: BLIMP-1 ↑ granzyme B expression ↑	Attenuating the antitumor response of cytotoxic T cells	In melanoma cell lines + murine models	[42]
/	miR-26b-5p and miR-21-3p ↑	TAP1 protein ↓ HLA class I cell surface antigens ↓	Decreasing the recognitions of T cells	Only in melanoma cell lines	[43]
/	miR-28 ↑	Targets: PD1, TIM3 and BTLA ↓ IL-2 and TNF-α ↑	Promoting exhaustive differentiation of T cells	In melanoma cell lines + murine models	[44]
/	miR-30b/d ↑	Target: GALNT7 ↓ IL-10 expression ↑	reduction of CD3^+^ T cells recruitment and induction of regulatory T cells.	In melanoma cell lines + murine models	[45]
/	miR-210 ↑	Targets: PTPN1, HOXA1, and TP53I11 ↓	Increasing susceptibility of tumor cells to lysis by cytotoxic T cells	Only in melanoma cell lines	[46]
/	miR-30c, miR-23a, miR-4299 ↑	Target: TNFRSF8 ↓ CD30 expression ↓	Associating of dysfunctional immune response	Only in melanoma cell lines	[47]
/	miR-498 and miR-3187-3p ↑	Targets: PTPRC (CD45) ↓ TCR signaling ↓ TNFa secretion ↓	Reducing the functions of CD8^+^ T cells	Only in melanoma cell lines	[48]
Hypoxic stress in melanoma cells and Cx43 signaling upregulation	miR-192-5p ↑	Target: ZEB2 ↓	Lowering cytotoxic activity of T cells	In melanoma cell lines + murine models	[49]
/	miR-155 ↑	Targets: STAT5, SHIP1, SOCS1, and PTPN2 ↓ Akt and Stat5 signaling ↓	Enhancing CD8^+^ T-cell antitumor responses	In melanoma cell lines + murine models	[49,50,51,52,53,54]

Cancer-associated fibroblasts (CAFs), the activated fibroblasts, are involved in tumor progression and metastasis as well as the initiation of cancer. Fibroblasts play a suppressing role in the development of early-stage melanoma. Research has shown that dermal fibroblasts inhibit melanoma growth in the early stage by causing cell cycle arrest and hindering the epithelial–mesenchymal transition [22]. However, with the growth of the tumor, fibroblasts are reprogrammed into CAFs to promote tumor progression [55]. Before the invasion of melanoma cells, some melanosomes are first released by melanoma cells into the dermis. Such melanoma-derived exosomes contain a high amount of non-coding RNAs which are biologically active. The uptake of those miRNAs by fibroblasts can result in the formation of CAFs [19,20]. For example, the high level of miR-211 in melanoma-derived exosomes directly decreases the expression of insulin-like growth factor 2 receptor (IGF2R) in fibroblasts, thus, upregulating the MAPK signaling pathway to induce the primary CAFs reprogramming [20]. Similarly, miR-210 and miR-155 delivered by melanoma-derived exosomes also trigger a metabolic reprogramming function in the fibroblasts [21]. Meanwhile, in BRAF-wildtype and BRAF-melanoma cell lines, miR-210 and miR-155 could promote glycolysis and inhibit oxidative phosphorylation in metabolic conditioning to trigger the reprogramming and the formation of CAFs. Moreover, miR-21 determines the TGF-β1-induced formation of CAFs in vitro and in vivo [19]. The over-expression of miR-21 directly decreases the expression of its target protein SMAD Family Member 7 (Smad 7), thus regulating TGF-β signaling to activate the formation of CAFs. In vivo, the overexpressing of miR-21 promotes melanoma tumor growth, whereas the knockdown of miR-21 blocks TGF-β1-induced ferroptosis suppressor protein 1 (FSP1) expression and CAFs formation. During the reprogramming, the angiogenesis of CAFs is also engendered. Zhou et al. (2018) discovered the role of miR-155-5p in the proangiogenic switch of CAFs by bioinformatics analysis, a luciferase reporter assay, and microRNA mimic and inhibitor and xenograft models [22]. The authors found that the exosomal miR-155 directly targeted SOCS1 gene which resulted in the proangiogenic switch of CAFs with increasing expressions of proangiogenic factors (VEGFa, FGF2, and MMP9) through the activation of JAK2/STAT3 signaling pathway [22].

Dendritic cells (DCs) are antigen-presenting cells (APCs), which can effectively recognize, acquire and present antigens to naive T cells. In addition, DCs also have interactions with other immune cells including B cells, NK cells and so on [56,57]. Thus, DCs served as a bridge between innate and adaptive immune systems. DCs develop directly from myeloid progenitors in the bone marrow (BM) [58,59]. After a series of differentiation events, progenitor cells evolve into immature DCs (imDCs) and then turn into mature DCs (mDCs) [59]. During the cell cycle of DCs, several critical miRNAs are involved in DCs differentiation, maturation, function and apoptosis. For example, Lu et al. (2011) found 27 miRNAs that had greatly different expressions between imDCs and mDCs, indicating their roles in the maturation of DCs [60]. Among them, miR-211 and miR-155 regulate the development and apoptosis of DCs as well as the expression of cytokine IL-12 through targeting p27kip1, KPC1, and SOCS-1 [60,61]. It has been discovered that miR-22 expressed in DCs could enhance the anti-tumor function of DCs [24]. As a direct target of miR-22, p38 is down-regulated and some DC-derived cytokines e.g., interleukin-6, are also decreased, which hinders the differentiation of DC-driven Th17 cells. Likewise, the overexpression of miR-128 in DCs can prevent the expression of p38, while also leading to reduced interleukin (IL)-6 and IL-10 cytokine levels and elevated IL-12 in DCs [26]. By targeting several signaling pathways and proteins, miR-27a, which is associated with the overexpression of TGF-β, could suppress the DC-dominated differentiation of Th2 and Th17 cells, thus enhancing melanoma cell growth [25]. Moreover, a recent study showed that the overexpression of miR-9 could activate DCs by targeting PCGF6, leading to the activation of CD4^+^ and CD8^+^ T cells [23]. MiR-192-5p and miR-148a-3p are delivered to DCs through the connexin 43 (Cx43) channel in hypoxic melanoma cells to inhibit the functions of immune cells [49].

The tumor-associated macrophages (TAMs) participate actively in the innate immune response to the early stage of melanoma. The TAMs contain two different phenotypes: the pro-inflammatory and tumoricidal M1 type, and the “alternative” anti-inflammatory and tumor-promoting M2 type. The differentiation between M1 and M2 macrophages can be modulated not only by proteins such as INFγ but also by miRNAs. For example, a miR-125 cluster is a pivotal regulator in polarization [30,31,32]. *Listeria monocytogenes* activates the expression of miR-125a-3p and miR-125a-5p, which negatively regulate host defense and bactericidal activity, while the expression of miR-125b-5p is modulated by NF-κB signaling. While one study in 2011 showed that miR-125b could promote inflammatory responses and has antitumor activities [62], a more recent study in 2020 has, however, reported the tumor-promoting properties for miR-125-5p [32]. MiR-125b-5p can be delivered to macrophages and catalyze the phenotype switch from M1 to M2 macrophages. Meanwhile, miR-21 is also known for its tumor-promoting role in the macrophages [28]. Studies have shown that miR-21 decreases the expression of JAK2 and STAT1 to suppress the IFN-γ-induced STAT1 signaling pathway, blocking the polarization activation of M1 macrophages [27,28]. In murine models, the miR-21 knockout mice developed smaller melanoma tumors than wild-type mice [27]. Sahraei et al. (2019) found that the absence of miR-21 also promotes the anti-tumor ability of cytotoxic T-cells by releasing cytokines and chemokines such as IL-12 and C-X-C motif chemokine 10 [28]. The study also found that melanoma tumor growth was inhibited by targeting miR-21, as it triggered an overall proinflammatory angiostatic function. Prominently, miR-146 is also a central macrophage-regulating miRNA. MiR-146a can decrease the production of RIG-I-dependent type I interferon (IFN) production through NF-κB signaling by targeting TNFR-associated factor 6 (TRAF6) and Il-1R associated kinase 1/2 (IRAK1/2), thus attenuating the differentiation and polarization of macrophages, regulating inflammatory responses against melanoma. Meanwhile, the levels of miR146a/b can be regulated by TLR-induced signaling pathways such as PI3K-JNK, NF-κB, and IRAK1-TRAF6-NFκB [30,33,34].

The innate immune response is also maintained by NK cells, the CD56^+^CD3^−^ large granular lymphocytes [63]. However, miR-34a/c, on the other hand, decreases the recognition capacity of NK cells and promotes tumor growth. Its expression is negatively correlated with UL16 binding protein 2(ULBP2) and NK group 2 member D (NKG2D) protein. NKG2D, together with its tumor-associated ligands, form one of the most important receptor–ligand systems for the recognition and suppression of tumor cells [38]. MiR-155 also positively regulates NK cells by upregulating NKG2D, IFN-gamma and granzyme B production [39]. Notably, the over-expressed miR-200a-5p was associated with shorter overall survival of melanoma patients. Overexpression of miR-200a-5p decreases the level of antigen processing 1 (TAP1), followed by the reduced expression of human leukocyte antigen class I (HLA-I) antigens, thus altering the NK cell recognition and shortening the overall survival of patients, since both TAP1 and HLA-1 are required components for immune recognition [40].

Myeloid-derived suppressor cells (MDSCs) are heterogeneous immature cell populations that acquire tumor-promoting roles by suppressing the immune response of T cells and promoting angiogenesis and metastasis of tumor cells [64]. Several miRNAs have been implicated in the expansion of MDSCs in melanoma and other tumors. Through targeting of PTEN and the subsequent activation of the Akt pathway, TGF-β1-induced miR-494 in MDSCs is able to enhance the expansion and function of MDSCs in tumor tissues [41]. In addition, a group of miRNAs (let-7e, miR-99b, miR-100, miR-125a/b, miR-155, miR146a/b) are involved in the polarization and functions of MDSCs, and subsequently inhibit T-cell functions through the overexpression of IL-6 and CCL2 [18].

In addition to innate immunity, many studies have shown close interactions between miRNAs and the adaptive immune system in melanoma. Studies have shown that miRNAs can regulate the activation of cytotoxic T lymphocyte cells (CTLs) in many ways. The antitumor effect of CTLs can be enhanced by miR-23a, which reduces cytokine secretion of granzyme B and IFN-γ by directly targeting BLIMP-1 [42]. Lazaridou et al. (2020) reported that miR-26b-5p and miR-21-3p in melanoma cells regulated the frequency of CD8^+^ T cell infiltration and recognition through the downregulation of TAP1 and HLA class I cell antigens [43]. A recent study reported that miR-498 and miR-3187-3p in melanoma-derived exosomes inhibited the cytotoxic effect of CD8^+^ T cells by targeting IFN-α, protein tyrosine phosphatases receptor type C (PTPRC) [48]. Furthermore, miR-155 promotes the immune response of effector CD8^+^ T-cells. The deficiency of miR-155 in CD8^+^ T-cells decreases the activation of T-cell immune response but increases the population of myeloid cells, resulting in lower antitumor immunity [50,51,52]. The high expression of miR-155 is correlated with more cytokine production and stronger CD8^+^ T-cell responses in melanoma. Among melanoma patients, it was observed that upregulation of miR-155 in effector memory CD8^+^ T-cells increases their frequencies in tumor-infiltrated lymph nodes and is associated with prolonged overall survival of melanoma patients [53]. Moreover, several miRNAs are induced by hypoxia, a typical tumor microenvironment stressor in melanoma. The induction of these hypoxia-induced miRNAs negatively regulates the activities of CTLs. For example, there is an inverse correlation between the hypoxia-induced miR-192-5p level and the cytotoxic activity of CTLs [49]. Moreover, miR-210 induced in the hypoxic region of the tumor suppresses the susceptibility of melanoma cells to CTL-mediated lysis [46].

In addition to cytotoxic T-cells, the function of regulatory T-cells can also be affected by miRNAs including miR-30c, miR-23a, and miR-4299 [45,47]. For instance, miR-28 was shown to promote the exhaustive differentiation of T cells by regulating PD1, TIM3, and BTLA [44]. The expression of miR-30b/d can also increase the production of IL-10 by targeting GALNT7, thus, reducing immune cell activation and recruitment [45].

### 2.2. MiRNAs in Immunotherapy

The advent of checkpoint inhibitor immunotherapy (CII) using immune checkpoint inhibitors (ICIs) to re-invigorate the immune system represents a breakthrough in melanoma treatment. It has significantly improved the outcomes of metastatic melanoma patients. Ipilimumab, which targets cytotoxic T lymphocyte-associated antigen 4 (CTLA-4), and nivolumab and pembrolizumab, which are inhibitors of programmed cell death protein-1 (PD-1), were approved by Food and Drug Administration (FDA) to treat metastatic melanoma. CIIs can achieve a more durable response in melanoma patients compared with BRAF-targeted therapy. However, even the optimal regimen with a combination of CIIs leaves half of the patients failing to achieve long-lasting benefits [65], highlighting the need to identify predictive biomarkers for assessing the outcomes and new therapeutic targets for overcoming resistance. Research has found that the expressions of miRNAs in blood or biopsy in melanoma patients are closely related to the outcome of immunotherapy, suggesting that miRNAs could serve as practical and inexpensive biomarkers for predicting the response rate or survival time of patients treated by immunotherapy. Table 2 summarizes the regulations of miRNAs in melanoma patients receiving ICI treatments including PD-(L)1 and CLTA-4 inhibitors.

**Table 2:** Regulation of miRNAs in immunotherapy of PD-(L)1 inhibitor/CLTA-4 inhibitors.

MiRNAs	Treatment	Regulation	Sample Size	Source	Literature
miR-155	PD-(L)1 inhibitor	miR-155 was upregulated after receiving anti-PD-1 treatment, which associated with prolonged overall survival	9 healthy donors and 13 patients	blood, tumor tissue	[53]
miR-100-5p and miR-125-5p	PD-(L)1 inhibitor	miR-100-5p and miR-125-5p were upregulated in responding patients, which associated with prolonged overall survival	13 no clinical benefit patients and 9 clinical benefit patients	biopsies	[16]
miR-16-5p, miR-14-5p, and miR-20a-5p	PD-(L)1 inhibitor	The levels of miR-16-5p, miR-14-5p, and miR-20a-5p was twice higher in the responding group than non-responding group	33 patients with malignant melanoma	serum	[17]
exo-miRNA-532-5p and exo-miRNA-106b	PD-(L)1 inhibitor	The levels of exo-miRNA-532-5p and exo-miRNA-106b significantly decreased in patients than in healthy people (Z = −4.17 and −4.57, respectively, *p* < 0.0001)	95 patients and 95 healthy donors	serum	[66]
miR-4649-3p, miR-615-3p, and miR-1234-3p	PD-(L)1 inhibitor	MiR-4649-3p, miR-615-3p, and miR-1234-3p were significantly decreased in completing-responding patients and partial-responding patients compared with the non-responding group	47 malignant melanoma patients	blood	[67]
miR-34c, miR-711, miR-641, and miR-22	CLTA-4 inhibitor	MiR-34c, miR-711, miR-641, and miR-22 were identified to be significantly associated with progression free survival (PFS) in advanced melanoma patients treated with neoadjuvant ipilimumab	27 melanoma patients	biopsies	[68]
miR-222	CLTA-4 inhibitor	The expression of hsa-miR-222 in melanoma tissues of clinical benefit patients was 2.3-fold higher (*p*-value = 0.001) than in no-clinical benefit patients	12 clinical benefit patients and 23 no-clinical benefit patients	tumor tissue	[69]

#### 2.2.1. The Involvement of miRNAs in Response, Resistance, and Side Effects of Immunotherapy with Immune Checkpoint Inhibitors

##### PD-(L)1 Inhibitor

Emerging evidence has highlighted the extensive involvement of miRNAs in immunotherapy in melanoma patients. Notably, the interaction of miR-155 with anti-PD-1 immunotherapy has been demonstrated in melanoma [53], lung cancer [70], and renal cell carcinoma [71]. The upregulation of miR-155 expression was observed in vivo and in situ after anti-PD-1 treatment, wherein the increased miR-155 expression led to the decreased expression of its target gene PTPN2. Indeed, melanoma patients have a high expression level of miR-155 after receiving anti-PD-1 treatment. The downregulation of miR-155 target signature due to the overexpression of miR-155 correlated with prolonged overall survival (OS) in melanoma patients [53]. In addition to the well-studied miR-155, the elevated level of some other miRNAs has been shown to indicate a positive response to PD-1 inhibitors. By analyzing the expression of a core miRNAs cluster between the clinical benefit and the no clinical benefit groups, the expressions of miR-100-5p (median log_2_ counts: 12.48 vs. 11.25, *p*-value = 0.036) and miR-125-5p (median log_2_ counts: 17.35 vs. 15.49, *p*-value = 0.025) were significantly higher in responding patients. It has also been shown that the expression of miR-100-5p and miR-125-5p positively correlated with the overall survival of melanoma patients treated with PD-1 inhibitors [16]. Nakahara et al. (2020) analyzed 2560 miRNAs in the serum samples from anti-PD-1 treated melanoma patients. The microarray analysis revealed that the levels of miR-16-5p, miR-14-5p, and miR-20a-5p were more than twice higher in the responding group than the non-responding group, suggesting that they could serve as valuable indicators of successful PD-1 inhibitors treatment [17].

Some miRNAs were found to be downregulated in PD-1 inhibitor-responding patients. A study compared exosomal miRNA expressions in the serum samples of melanoma patients who were treated with pembrolizumab with those of healthy individuals. The results showed that exo-miRNA-532-5p and exo-miRNA-106b were dramatically decreased after pembrolizumab treatment compared to the control group (Z = −4.17 and −4.57, respectively, *p* < 0.0001) [66]. Measuring the levels of miR-532-5p and miR-106b to distinguish melanoma patients from healthy individuals obtained over 88% accuracy in blinded tests of 50 mixed serum samples. This demonstrates the potential of miRNAs in clinical diagnosis. Another study examined the presence of 2083 miRNAs among complete-responding, partial-responding, and non-responding melanoma patients receiving PD-1 inhibitors. The study showed that the expressions of miR-4649-3p, miR-615-3p, and miR-1234-3p were significantly decreased in completing-responding patients and partial-responding patients compared with the non-responding group, indicating that these miRNAs negatively correlates with response outcome of PD-1 inhibitor treatment [67].

##### CLTA-4 Inhibitor

Ipilimumab, a CTLA-4 inhibitor that was approved by FDA in 2011, was the first ICI to treat advanced unresectable melanoma due to its clinical benefit [72]. A 4-miRNAs signature consisting of miR-34c, miR-711, miR-641, and miR-22 was identified to be significantly associated with Progression Free Survival (PFS) in advanced melanoma patients treated with neoadjuvant ipilimumab [68]. MiR-222 had a significantly higher expression level (2.3-fold higher, *p*-value = 0.001) in the tissues of the patients who responded to ipilimumab than non-responders. Therefore, miR-34c, miR-711, miR-641, miR-22, and miR-222 could be potential biomarkers of the response to anti-CTLA-4 treatment [69].

Clinical response to immunotherapy is limited, with about 15% of patients responding to CLTA-4 inhibitors [73]. A plausible explanation is that additional independent immune suppressive pathways facilitate T-cell exhaustion and tumor escape [74]. Therefore, targeted inhibition of multiple immune suppressive pathways is required to achieve a synergistic anti-cancer effect [75,76].

##### Combination Treatment of Immune Checkpoint Inhibitors

Increasing evidence has shown that the combination of ipilimumab (CTLA-4 inhibitor) and nivolumab (PD-1 inhibitor) achieved better long-term outcomes in treating melanoma [77]. In a recent study of metastatic renal cell carcinoma, several miRNAs were found to be correlated with response to the combination treatment of ipilimumab and nivolumab [71]. The high expressions of miR-138, miR-155, miR-200b, and miR-211 were detected in the non-responding patients, while up-regulated miR-200a and miR-497 were found in the responders. Even though the combined use of CTLA-4 inhibitors and PD-1 inhibitors has shown significantly more satisfactory clinical outcomes in melanoma than either alone, little research has been done to identify the miRNA profile signatures that feature the varied clinical outcomes of the combined therapy of ICIs.

##### Other Immune Checkpoint Inhibitors

LAG3, the gene product of lymphocyte-associated gene 3, is a transmembrane protein expressed on immune cells [77]. LAG3 is an inhibitory immune checkpoint contributing to blocked immune response and T-cell exhaustion [78]. A clinical trial showed that LAG3-blocking antibody has synergistic effects with conventional immune checkpoint inhibitors for melanoma. Melanoma patients treated with the combination of anti-LAG3 antibody Relatlimab and anti-PD-1 antibody Nivolumab have median progression-free survival of 10.1 months compared with 4.6 months with Nivolumab alone [79]. Therefore, miRNAs that regulate the expression and function of LAG3 and its immune suppressive pathway have promising immunotherapeutic potential. However, limited work has been conducted in this research area, with only miR-15a and miR-16 being discovered to engage in regulating LAG-3. The deficiency of miR-15a and miR-16 leads to a low expression of LAG-3 in CD8^+^ T [80]. Identification of miRNAs involving in regulating LAG-3-mediated immune suppressive pathways and the mechanisms of how LAG3 synergizes with other immune checkpoint inhibitors deserve further exploration.

In addition, CD73 is another promising immune checkpoint target for viable immunotherapy. CD73 represents an ecto-5′-nucleotidase which is expressed on the surface of multiple types of cells including lymphocytes, hydrolyzing extracellular adenosine monophosphate (AMP) into adenosine (ADO) and inorganic phosphate [81]. CD73 modulates the immune response, immune escape, tumor metastasis, microenvironment, and drug resistance [82,83,84]. High expression of CD73 is associated with poor prognosis in melanoma patients [85,86]. Targeted blockage of CD73 induces an antitumor immune response and synergizes with other immune checkpoint inhibitors, especially PD-1/PD-L1, to promote the anti-melanoma effect [87]. Only a few miRNAs have been identified to regulate CD73 directly. For instance, the miR-30 family can decrease cell proliferation, migration, and survival by targeting CD73 in colorectal cancer and gallbladder carcinoma [88,89]. Similarly, through regulating CD73, the overexpression of miR-340 significantly inhibited cell proliferation, migration, and survival in gallbladder carcinoma [89]. Lower miR-422a levels are correlated with high CD73 expression and shorter survival time in neck squamous cell carcinoma patients [90]. In addition, miR-187 directly targets CD73 in vitro in colon cancer cell lines [91], and miR-193b expression negatively correlates to the CD73 protein level in pancreatic cancer [92]. However, the involvement of miRNAs in the expression and immune suppressive roles of CD73 in melanoma has not been studied and represents a promising new research direction.

##### Involvement of miRNAs in Drug Resistance against Immune Checkpoint Inhibitors

MiRNAs are active players in drug resistance to targeted therapy in melanoma. Several miRNAs including miR-146a, miR-204-5p, miR-211-5p, miR-550a-3-6p, miR-199b-5p, miR-126-3p, and miR-204-5p have been proven to contribute to melanoma resistance to BRAF inhibitors or/and MARK inhibitors [93,94,95,96,97]. However, the miRNA-mediated resistance to immunotherapy in melanoma has not been sufficiently studied and remains poorly understood. Nonetheless, a volume of evidence shows that a high frequency of MDSCs could represent an underlying mechanism of resistance to cancer immunotherapy [98,99,100]. Since miRNAs intricately interact with MDSCs, manipulating the expressions of MDSCs-related miRNAs may alter the melanoma cells’ sensitivity to ICIs. Indeed, a set of miRNAs (miR-164a, miR-155, miR-125b, miR-100, let-7e, miR-125a, miR-146b, miR-99b) was found to be accumulated in the plasma of melanoma patients. The plasma is collected from 20 melanoma patients with advanced disease (stage IIIC unresectable and stage IV) and from a group of age- and sex-matched healthy people (n = 20). These MDSC-miRs that are positively associated with MDSCs can help melanoma cells gain resistance against ICIs treatment, and thus serve as potential biomarkers for predicting durable response [18].

##### Role of miRNAs in Side Effects of Immune Checkpoint Immunotherapy

One of the major disadvantages of immune checkpoint immunotherapy is that the use of immune-checkpoint inhibitors can cause profound immune-related adverse events (irAEs). Such adverse effects can vary in different melanoma patients but are generally characterized by dermatologic toxicity, gastrointestinal toxicity, endocrinopathies, and pneumonitis [10]. High-grade irAEs can compromise life quality and are even life-threatening, and are the primary cause for the termination of immunotherapy [9].

In previous sections, we discussed the regulatory functions of miR-146a in immune cells. In the murine model, Marschner et al. (2020) found that miR-146a knockout mice developed more irAEs when treated with immune checkpoint inhibitors. The miR-146a-deficient mice manifest enhanced activator and effector function of T cells upon ICI treatment and a dramatic increase in neutrophil in the spleen and inflamed intestine. Clinically, the irAE severity was confirmed in 167 patients. It was concluded that the decreasing expression of miR-146a corresponded to an increased risk of irAEs and shorter survival among ICI-treated melanoma patients [101].

#### 2.2.2. MiRNAs Associated with Other Immunotherapies

##### Tumor-Infiltrating Lymphocytes (Tils)

As early as 1969, tumor-infiltrating lymphocytes (TILs) were first characterized in the control of melanoma [102]. Nowadays, the correlation of TILs with survival rates of melanoma patients has been widely verified [103,104]. After isolating and ex vivo expansion, autologous tumor-infiltrating lymphocytes (TILs) are infused with IL-2 into patients, a process known as adoptive cell therapy [35]. The outcomes of TIL trials in metastatic melanoma patients showed a durable response [35,105].

A recent study analyzed miRNA expression patterns in melanoma patients who received TILs adoptive cell therapy [106]. A significantly higher expression of miR-34a-5p and miR-22-3p was detected in TILs-responding patients than in non-responders. The patients who had over-expressed miR-34a-5p corresponded with improved survival time. In vitro experiments illustrated that miR-34a-5p and miR-22-3p inhibited T-cell cytotoxicity [106].

##### Toll-like Receptor 9 Agonist

Toll-like receptor 9 (TLR-9) is a member of the toll-like receptor (TLR) family. TLR9 is a receptor expressed in immune cells including natural killer cells, macrophages, dendritic cells, and other antigen-presenting cells [107]. TLR-9 triggers signaling cascades leading to a cytokine response, which subsequently activates a broad range of immune cells [108]. The expression of TLR-9 is diminished in breast cancer and renal cell carcinoma, and higher expression of TLR-9 corresponds with better clinical outcomes [109]. Lately, TLR-9 agonists have drawn considerable attention at the frontier of research in melanoma immunotherapy since it alters the tumor microenvironment and enhances the anti-tumor immune response by elevating the expression of the TRL-9 receptor [110].

While drugs such as Viautolimod (CMP-001), Lefitolimod, and Tilsotolimod are still in the early stages of clinical trials, they have already presented encouraging results to overcome the drug resistance of immune checkpoint inhibitors in advanced melanoma. For instance, recent studies have proved that Viautolimod could help maintain persistent responses when combined with pembrolizumab in melanoma patients [111,112,113]. The durable performance of Lefitolimod was also found in combination therapy with ipilimumab against drug resistance [114], and so as for Tilsotolimod [115]. In addition, studies have demonstrated that several miRNAs, such as miR-17~92 families, miR-126, and miR-223, are involved in the activation and signaling pathway of TLR-9 receptors [116,117,118]. In vivo and in vitro, TLR-9 signaling was found to repress miR-7 expression to strengthen the anti-tumor effect [119,120]. All these miRNAs are potential biomarkers for the outcome of TRL-9 agonist treatment or targets for developing combined immunotherapy with TRL-9 agonists.

## 3. MiRNAs in Immunity and Immunotherapy of Uveal (Eye) Melanoma (UM)

The role of immune-related miRNAs in uveal melanoma (UM) has been investigated in several studies. MiRNAs directly or indirectly interact with immune cells in the microenvironment of uveal melanoma (UM). A cluster of miRNAs (miR-20a, miR-146a, miR-155, miR-181a, miR-223) were reported to regulate NK cells, and desensitize UM cells to NK cell cytolysis [121]. Additionally, a population of immune cells (including circulating T-cells, natural killer cells, natural killer T-cells, and myeloid cells) and immune regulatory miRs (miR-20a, miR-125b, miR-146a, miR-155, miR-181a, and miR-223) were assessed in six UM patients [122]. Alterations in immune-related miRs were observed in the cell populations of CD3^+^ natural killer T-cells, CD56^+^ natural killer T-cells, and CD15^+^ myeloid suppressor cells. Additionally, miRNAs can modulate immune-regulated cytokines to change the tumor microenvironment in UM. As discussed in the previous section, the overexpression of miR-21/22/29a and the down-expression of miR-30b/30d/128 can decrease the secretion of IL-10, and thus moderates immune response (see Table 2). A study found that IL-10Rα expression is directly regulated by miR-15a, miR-185, and miR-211 in the UM cell. The upregulation of miR-15a, miR-185 and miR-211 can decline the expression level of IL-10Rα [123].

## 4. Novel miRNAs-Based Immunotherapy

As discussed in previous sections, the expressions of some miRNAs correlate with the therapeutic outcomes of ICIs, suggesting that the combination of miRNAs and ICIs can be a viable therapeutic strategy that improves the efficacy and outcomes of ICSs. In vivo, a study illustrated that combination therapy of miR-146a antagomiR (anti-miR-146a) and anti-PD-1 antibody exhibited better performance in shrinking melanoma tumors and improving survival rate compared to solo anti-PD-1 treatment [124]. Another study has found that, after receiving PD-1 antibodies, the mice with miR-21-deficient macrophages developed smaller melanoma tumors than the control group [27]. These research findings suggest that miRNA could be a promising immunotherapy agent which deserves further exploration.

MiRNAs are intricately implicated in both innate and adaptive immunity, inferring that miRNAs can potentially combat melanoma by sensitizing or re-invigorating the immune responses that target melanoma. MiR-34a is a naturally occurring tumor suppressor miRNA whose expression is absent or reduced in an array of cancers [125]. MiR-34a downregulates the expressions of more than 30 oncogenes such as BCL2, CDK4/6, CD44, NOTCH1, MYC, MET, and PDGFR-α [126], and genes involved in tumor immune escape including PD-L1 and DGKζ [127,128,129]. MRX34, a liposomal formulation of the miR-34a, is the first-in-class miRNA-based therapy for various cancers including melanoma [130,131]. Even though MRX34 administration achieved partial response in isolated cases previously heavily treated with resection and PD-1 inhibitor, the clinical phase 1 trial of MRX34 for melanoma was terminated due to the development of serious irAEs such as seizure and disobedient mentality [131,132]. The current setback of MRX34 treatment for melanoma highlights the increasing need to seek more miRNA candidates that are potential targets for effective immunotherapy. At the same time, an efficient and safe delivery system of miRNA-based immunotherapy has to be developed, which is discussed in the next section.

## 5. The Delivery Strategy of miRNA-Associated Immunotherapy

Since tumor suppressor miRNAs retard tumor development and modulate anti-cancer immunity, targeted delivery of miRNAs to cancer cells is becoming an attractive option for melanoma treatment [133]. Nanoparticles used as drug delivery carriers exhibit unique properties and have the potential to improve the therapeutic efficacy of anti-cancer agents [134]. The development of miRNA-based gene therapy using nanotechnology may overcome the therapeutic barriers and stabilize the miRNA to prolong its circulation in the bloodstream [135]. Nanoparticles can be modified to deliver the miRNA molecule specifically to the target site in a sustained and controlled manner, and therapeutic outcomes could be significantly improved [136]. This section surveys the potential nano-formulations that can be used in melanoma miRNA immunotherapy.

### 5.1. Lipid Nanoparticles

The lipid-based nanoparticle is a class of drug delivery carrier system that can successfully transport poorly water-soluble drugs and oligonucleotides in gene therapy. The cationic lipid-based nanoparticle was first used to encapsulate miRNA. It can improve transfection efficiency by facilitating cell membrane interaction [137]. The lipid-based delivery systems have the potential to target cancer that has metastasized to the brain. Even without any surface functionalization, their small size and ability to cross the blood–brain barrier make them an excellent treatment candidate for central nervous system disorders [138]. Meanwhile, they can be modified with active targeting ligands to achieve specific targeting [132]. Based on the structures, lipid-based nanoparticles can be subdivided into liposomes, lipid nanoemulsions, solid lipid nanoparticles, lipid nanoparticles, and nanostructured lipid carriers.

Lipid-based nanoparticles have been used to encapsulate miR-34a for various cancer treatments [135]. A liposome–polycation–hyaluronic acid (LPH) nanoparticle was formulated to deliver miR-34a for melanoma treatment. This delivery platform could significantly improve the therapeutic effect and inhibit lung metastasis [136]. Additionally, miR-34a and paclitaxel were co-delivered by a cationic lipid nanoparticle for synergistic melanoma therapy. This promising delivery system eliminated the mice model’s lung metastasized cancer cell population [139]. A phase I study of a liposomal miR-34a mimic, MRX34, was studied in patients with advanced solid tumors. However, this first-in-human clinical trial was terminated due to an unexpectedly severe immune-mediated adverse event, which resulted in four patient deaths [131]. Another phase I pharmacodynamics study of MRX34 in melanoma patients was withdrawn because five immune-related adverse severe events occurred [140]. Therefore, an effective delivery strategy is still in urgent need, and active targeting is preferred to minimize systemic immune activation.

### 5.2. Silica Nanoparticles

Inorganic nanoparticles are attractive drug delivery candidates and have been extensively studied over several decades. One of the most promising inorganic materials for biomedical applications is mesoporous silica [141]. Mesoporous silica nanoparticles have a porous structure that can encapsulate various therapeutic agents. Meanwhile, they have a large surface area that allows further modification for targeted delivery [142]. Rosa et al. (2020) successfully loaded miR-34a-3p into a silica-based nanoparticle. The hollow silica nanoparticle was modified with spermidine to target human melanoma cells that over-expressed the polyamine transport system. The strategy significantly promoted cellular uptake and induced apoptosis [143]. Furthermore, Yang et al. (2021) used functionalized mesoporous silica nanoparticles loaded with miR-125a to create a tumoricidal environment by enhancing immune response and reversing immunosuppression. It was observed that the intratumor injection enhanced the infiltration of natural killer and CD8^+^ T-cells within the tumor. Meanwhile, due to the repolarization of tumor-associated macrophages, this may be a helpful tool to initiate antitumor immunity, potentially inhibiting tumor growth in mouse models in a cooperative manner. Therefore, this formulation may serve as an effective potential agent for cancer immunotherapy [133,144].

### 5.3. Gold Nanoparticles

Metal-based nanoparticles also can be used for miRNA delivery [145]. The gold nanoparticle is another attractive material for nucleic acid delivery. Gold nanoparticles can be fabricated and modulated by regulating particle size and surface functionality, allowing covalent attachment of the nucleic acids. Alternative to the covalent system, the highly negatively-charged nucleic acid can be noncovalently absorbed on a cationic gold nanoparticle’s surface by a self-assembly [146].

A gold nanoparticle was used as a carrier to deliver siRNAs for uveal melanoma treatment. Four miRNAs (miR-34a, miR-182, miR-137, and miR-144) were chosen because they were found to be downregulated in uveal melanoma cells. This combination reprogrammed the cancer cell and restored normal behavior. The miRNAs were conjugated to the gold nanoparticle to overcome the inherent limitations of biomedical applications. This delivery platform increased Mel 202 cells’ sensitivity to SN38 (7-ethyl-10-hydroxy camptothecin), a topoisomerase I inhibitor [147].

### 5.4. Extracellular Vesicles

Extracellular vesicles (EVs) are lipid-bound particles that are naturally released from almost all types of cells [148]. EVs secreted by cancer cells protect and maintain the growth of cancer cells. They play an important role in anti-cancer drug-resistant development [149]. On the other hand, EVs released by normal cells, such as mesenchymal stem cells, may inhibit tumor growth and suppress cancer progression [150]. The content of EVs includes proteins, DNA, mRNA, and miRNA. They offer a novel therapeutic delivery method for cancer treatment. By apoptotic signal mediation, EVs modulate immune cells or immature precursors during melanoma migration. Meanwhile, EVs play an important role in promoting cancer cell escape from immune surveillance [151].

A tumor-targeted blood exosome nanoplatform was engineered for gene and chemo combination therapy. The delivery platform was loaded with doxorubicin and cholesterol-modified miRNA21 inhibitor (miR-21i). In vitro studies showed that cargo delivery efficiency was maximized, and both cellular accumulation and endosome escape ability of the payload were improved. The nanoplatform significantly enhanced tumor suppression in the tumor-bearing animal model, and no noticeable side effects were observed [152].

### 5.5. Dendrimer

Dendrimers are synthetic, continuously branched nano-sized polymeric macromolecules. They have tailored functionalized end groups that can be used for conjugating therapeutic agents and targeting moieties. Dendritic macromolecules exhibit narrow molecular weight distribution, tunable size, and shape. It has been considered an ideal delivery platform in drug formulation and nanopharmaceuticals development. As a delivery carrier, dendrimer can improve drug water solubility, enhance formulation stability, prolong blood circulation half-life and decrease immunogenicity [153].

Cationic dendrimers are promising miRNA carriers due to their unique properties [154]. Polyamidoamine (PAMAM) is one of the most frequently used dendrimers in gene therapy owing to its synthesis simplicity and has been produced on an industrial scale [155]. The classical terminal groups of PAMAM are amino groups. They are available for nucleic acid binding and targeting moiety conjugation [156]. A PAMAM dendrimer-based nanoparticle was formulated to deliver the miRNA mimic let-7b. Hyaluronic acid was used to target CD44, and chloroquine was employed to aid the endosomal escape. This delivery platform enhanced cellular uptake and cytotoxicity. After the treatment, both KRAS and BCL-2 gene expression were decreased, and p-21 expression was increased [157]. In addition, Maghsoudnia et al. (2020) introduced the triphenylphosphonium cation as a targeting moiety for the PAMAM delivery system. This strategy improved cellular uptake and mitochondria-targeted delivery of let-7b miRNA mimic [158].

### 5.6. Future Challenges

Nanotechnology holds great promise for melanoma miRNA treatment [159]. However, the working mechanisms of nano-biosystems are still unclear, and their predictive capabilities are limited [160]. Meanwhile, the therapeutic application of nanotechnology is hampered by several factors. For example, EVs, as noted above, lack an efficient and reproducible miRNA loading method. In addition, the enhanced permeability and retention effect worked in mouse models but not in humans, and an active targeting strategy is required to avoid the off-target effects [161]. Furthermore, the potential of immunogenicity is also a challenge in this field. Several miRNA liposome formulation clinical trials were withdrawn due to the unexpectedly severe immune-mediated adverse events. After internalization by the target cell, the endosome is considered an additional barrier. A lysosomotropic agent is preferred for helping miRNA escape from the endosomes to avoid the endolysosomal degradation pathway [162]. Future research should focus more on developing an active delivery platform and elucidating their molecular mechanisms in nanopharmacology.

## 6. Conclusions

Immunotherapy introduces a novel concept and strategy for treating cancers by unleashing immune checkpoint blockage, and it revolutionizes cancer treatment and management. Melanoma is a model malignancy that has benefited the most from immunotherapy. Immune checkpoint inhibitors have achieved impressive sustained response and improved the overall survival in melanoma patients compared with conventional chemotherapy and MAPK pathway targeted therapy. MiRNAs are highly conserved short non-coding RNAs that post-transcriptionally regulate gene translation. MiRNAs are profoundly involved in the differentiation, activation, and immune response of immune cells of innate and adaptive immunity, including macrophages, CAFs, DCs, NKs, MDSCs, and cytotoxic and regulatory T-cells. MiRNAs can also modulate the tumor microenvironment by affecting the expression of inflammatory factors including cytokines IFN-γ. MiRNAs interact with tumor suppressive pathways and thus have a significant impact on the therapeutic outcomes of ICIs, as well as on the irAEs and development of resistance to ICIs. As in cutaneous melanoma, miRNAs are implicated in the tumor immunity and immunotherapy of uveal and mucosal melanoma. The delivery of tumor-suppressive miRNAs is the key to the success of miRNA-based immunotherapy against melanoma. A variety of nanotechnology delivery strategies, such as lipid nanoparticles, silica nanoparticles, gold nanoparticles, extracellular vesicles, and dendrimers, have been developed. However, several clinical trials of nanoparticle formulation were withdrawn due to severe irAEs. An active targeting delivery strategy needs to be developed to achieve efficient loading and enhanced retention and avoid endolysosomal degradation and off-target effects.

This article is the first review that comprehensively integrates the roles of miRNAs in modulating the immunology of melanoma and in regulating immune checkpoint inhibition and other immune suppressive mechanisms. This review therefore highlights the prospect of miRNAs as promising immune modulation targets for developing combined immunotherapy with other immune checkpoint inhibitors.

## Data Availability

Not applicable.
